# Characterization of Chinese Unifloral Honeys Based on Proline and Phenolic Content as Markers of Botanical Origin, Using Multivariate Analysis

**DOI:** 10.3390/molecules22050735

**Published:** 2017-05-17

**Authors:** Ya-Qin Wen, Jinzhen Zhang, Yi Li, Lanzhen Chen, Wen Zhao, Jinhui Zhou, Yue Jin

**Affiliations:** 1 Institute of Apicultural Research, Chinese Academy of Agricultural Sciences, Beijing 100093, China; wenyaqin1219@163.com (Y.-Q.W.); jzzhang76@sina.com (J.Z.); liyi01@caas.cn (Y.L.); chenlanzhen2005@126.com (L.C.); zhaowen1309@126.com (W.Z.); 2 Bee Product Quality Supervision and Testing Center, Ministry of Agriculture, Beijing 100093, China; 3 Laboratory of Risk Assessment for Quality and Safety of Bee Products, Ministry of Agriculture, Beijing 100093, China; 4 Key Laboratory of Bee Products for Quality and Safety Control, Ministry of Agriculture, Beijing 100093, China

**Keywords:** botanical origin, phenolic compounds, proline, chemometrics, unifloral honeys

## Abstract

The phenolic and proline content were determined in honey samples of different floral origins (rapeseed, sunflower, buckwheat and *Codonopsis*) from five different regions of China. The phenolic and proline profile of these samples were used to construct a statistical model to distinguish honeys from different floral origins. Significant differences were identified among the studied honey samples from multivariate chemometric methods. The proline content varied among the four types of honeys, with the values decreasing in the order: buckwheat > *Codonopsis* > sunflower > rapeseed. Rapeseed honeys contained a high level of benzoic acid, while rutin, *p*-coumaric acid, *p*-hydroxybenzoic acid were present at relatively high levels in buckwheat honeys. Principal component analysis (PCA) revealed that rapeseed honey could be distinguished from the other three unifloral honeys, and benzoic acid, proline and kaempferol could serve as potential floral markers. Using 18 phenolic compounds and proline the honey samples were satisfactorily classified according to floral origin at 94% correct prediction by linear discriminant analysis (LDA). The results indicated that phenolic compounds and proline were useful for the identification of the floral origin of the four type honeys.

## 1. Introduction

Honey is popular for its nutritional and medicinal values. As a natural sweetening agent, honey is consumed directly, widely applied in the food industry, and also used as a food preservative [1]. Recent studies demonstrate that honey possesses many health benefits, including antimicrobial and anti-inflammatory effects, and heart disease and cancer risk reduction [2,3]. Honey has a complex composition consisting of a high concentration of sugars combined with minerals, free amino acids, enzymes, vitamins, phenolic compounds and numerous volatile compounds [4]. These components highlight both physical properties and nutraceutical characteristics of the product itself [5]. Different unifloral honeys may have different functional properties due to their different constituents.

In general, unifloral honeys is regarded as more valuable due to their good quality and pure flavour. Hence, market prices are determined by its botanical origin, and the increased value of some honey types stimulates the adulteration of honey of certain botanical origins. Honey adulteration, not only defrauds consumers, but also has impact on a country’s bee product exports. Therefore, identification of unifloral honey has attracted great attention from researchers. Identification of honey floral makers by analyzing of the physicochemical parameters like sugar content, diastase activity, amino acids or phenolic acid, flavonoids or volatiles have been used on certain unifloral honeys [6,7].

Phenolic compounds, mainly phenolic acids and flavonoids, comprise one of the most important constituents of honey, and have been considered as indicators of its antioxidant activity. The honey phenolic compounds originate as plant secondary metabolites, and their content in plants varies according to the plant species, variety, physiological stage, and environmental factors such as climate [4]. Phenolic compounds have been used to determine the botanical origin of unifloral honeys [8,9,10,11]. In previous research studies, several phenolic compounds have been reported to serve as floral origin markers for different unifloral honeys. For example, quercetin was suggested as a floral marker for sunflower honey [12]; gallic acid was the main phenolic acid in Manuka honey [13]; chlorogenic acid and ellagic acid were possible markers of acacia and rapeseed honeys, respectively [14]; ferulic acid, morin and kaempferol could distinguish chaste honey from rapeseed honey [15].

Proline is dominant amino acid in honey, and has been considered an indicator of honey quality [16]. The proline content in honey depends on the time the nectar is processed by the bees. Indirectly, proline levels also reflect botanical origin [17]. Previous studies found that the proline content of honey was associated with its floral and geographical origin [18]. Some studies indicated that sunflower honey contained slightly higher proline levels, as compared to rapeseed and acacia honeys [18,19].

China has a long history of beekeeping due to its vast geography, appropriate climatic conditions, and rich abundance of several plant species. There are a variety of honey types produced in China. The most common types of honey in China are rapeseed, acacia, *Vitex*, and jujube honeys. Other special types of honey, such as buckwheat, sunflower, and *Codonopsis*, are used mainly for commercial purposes. Even though China is one of the world’s largest honey exporters, the chemical composition of Chinese honey has not been comprehensively investigated. Previous studies on Chinese unifloral honeys are focused mainly on minerals, amino acids, and phenolic content [11,14,20,21,22], however, no report data on the phenolic and proline profile of certain Chinese unifloral honeys, such as sunflower and *Codonopsis*.

For these reasons, the principal aim of the present research was to investigate the proline and phenolic composition of four different unifloral honeys (rapeseed, sunflower, buckwheat and *Codonopsis* honey) collected from different regions in China. In addition, chemometric methods were applied to identify potential floral markers of different honey samples using phenolic compounds and proline as variables. These results provide insight into the composition of major phenolic compounds and proline profile of Chinese honeys and could help improve the market perception of Chinese honeys by lending credibility to their claims of authenticity.

## 2. Results and Discussion

### 2.1. Proline Profile of the Different Unifloral Honeys

**Figure 1:**
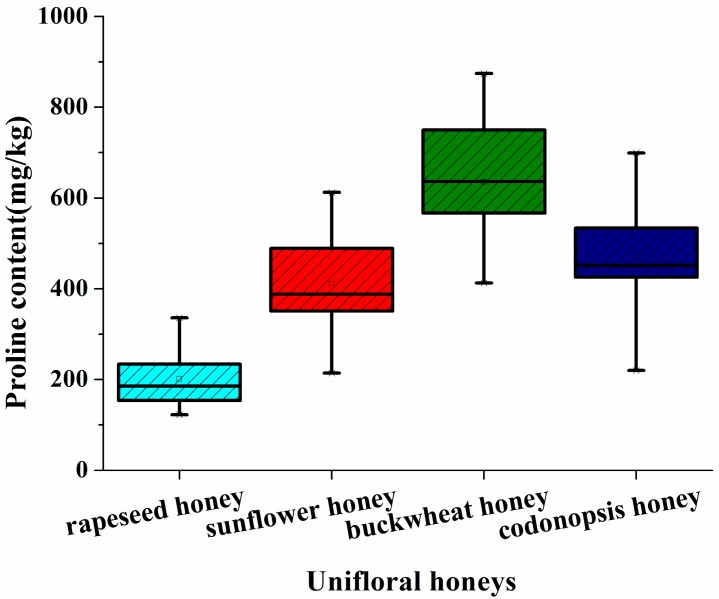
Box plot diagram of proline content in rapeseed, buckwheat, sunflower, and *Codonopsis* honey samples. The horizontal lines in the box denote the 25th, 50th, and 75th percentile values. The error bars denote the 5th and 95th percentile values. The height of the box is the measure for the tolerance.

The measured proline content of the different floral origin honeys is shown in Figure 1. Of the four unifloral honey species, the buckwheat honeys exhibited the highest proline content (average 610.16 mg/kg) (*p* < 0.05), followed by *Codonopsis* honeys (494.49 mg/kg), sunflower honeys (400.75 mg/kg), and rapeseed honeys (201.61 mg/kg). The observation that the proline content varied with the type of unifloral honey is consistent with previous analysis of European and Serbian unifloral honeys [18]. Our results confirmed that rapeseed honey exhibits low proline levels [17,18]. In addition, we observed large proline variation for different unifloral honey samples, which result from regional differences in the floral sources. For example, the range of proline content for rapeseed honeys was from 122.50 to 336.02 mg/kg, the sunflower honeys from 214.06 to 601.11 mg/kg, the buckwheat honeys from 412.56 to 874.62 mg/kg, and *Codonopsis* honeys from 380.37 to 699.53 mg/kg. These results are in accordance with other reports of variance in proline levels samples from unifloral honeys with different geographical sources [21,23]. The proline content of the three multifloral honey samples (S17, C10, C11) was 612.55, 497.74 and 219.77 mg/kg, separately (Supplementary Table S1). Although proline levels have been considered as a useful parameter for unifloral honey classification, the observed variance suggests that discrimination with proline is not complete and this measurement should be used together with other parameters including sugar and mineral content and the levels of phytochemicals [24]. Proline content in honey has been proposed as an indicator of honey quality [16], and this significant difference in proline content for different botanical types of honey may suggest variation of quality.

### 2.2. Phenolic Compounds in Four Different Honey Types

The distribution and levels of eighteen phenolic compounds in investigated honey samples were listed in Table 1. The phenolic content of the three multifloral honey samples (S17, C10, C11) was showed in Supplementary Table S1. Phenolic acids including protocatechuic acid, caffeic acid, gallic acid, *p*-coumaric acid, *p*-hydroxybenzoic, ferulic acid and benzoic acid, and flavonoids such as quercetin, kaempferol, pinocembrin, caffeic acid phenethyl ester (CAPE), chrysin and rutin showed differing amounts in the four types of unifloral honeys. Morin, myricetin, and naringenin were only detected at relative high level in rapeseed honey. Galangin were detected in rapeseed and sunflower honey. Morin was previously found in rapeseed honey by Zhou et al. [15]. Apigenin and CAPE were detected at low levels in the four unifloral honeys. Our analysis identified the predominant phenolic compounds for the four unifloral honeys as *p*-hydroxybenzoic, *p*-coumaric, caffeic, ferulic, and protocatechuic acid. The dominant flavonoids identified were quercetin, kaempferol, chrysin. These findings are in agreement with previous reports [25,26,27].

The levels of individual compounds can be strongly affected by floral and geographical origin. For example, gallic acid was found at the highest concentrations in *Codonopsis* honeys compared to the other three types of honey (*p* < 0.05). The amount of gallic acid found in sunflower honey was lower than that reported for Serbian sunflower honey [28]. The amounts of *p*-hydroxybenzoic acid and *p*-coumaric acid determined in our buckwheat honey samples were higher as compared to those of previous studies [25,29]. The determined amount of *p*-hydroxybenzoic acid was lower than that reported for buckwheat honey from the Shanxi Province in China [30], possibly reflecting regional differences of floral sources. The highest level of benzoic acid was found in rapeseed honeys. The average content of benzoic acid in rapeseed honey (9.475 mg/kg) was dozens of times higher compared to the levels in sunflower honey, buckwheat honey, and *Codonopsis* honey (*p* < 0.05). Heather honey has been reported to contain a high level of benzoic acid, thought to contribute to the aroma of heather honey [31]. The highest average content of caffeic acid and ferulic acid were found in sunflower honey (*p* < 0.05). Rutin (flavonol 3-*O*-rutinoside), which was presumed to be responsible for antioxidant activity of buckwheat [32], was detected at the highest levels in buckwheat honey (0.225 mg/kg), compared to the other three types of honeys. Rutin has also been detected in Polish rapeseed and multi-flower honeys [29], but was not found in studies of buckwheat honeys [25,33]. Rutin has never been found in buckwheat honeys, with the explanation that the phenolic pattern of the buckwheat nectar, and the corresponding honey, might be quite different from that of other plant tissues [25,33]. However, rutin was detected in our buckwheat honey samples for the first time, the reason may because of the variety and regional differences in floral sources, or the analysis method differences. The phenolic compounds analysis method (chromatography-multiple reaction monitoring-MS method) was validated by multiple parameters [15]. Chrysin has been found in many types of honeys including rapeseed honey and multifloral honeys [34], and here we found high levels of chrysin in sunflower honey and buckwheat honey.

More specifically, the phenolic content showed a great variability among different honey samples, for example caffeic acid ranged from 0.004 to 0.872 mg/kg in buckwheat honey, *p*-coumaric acid ranged from 0.002 to 0.051 mg/kg in *Codonopsis* honey, quercetin ranged from 0.022 to 5.184 mg/kg in rapeseed honey, and 0.002 to 0.659 mg/kg in sunflower honey. High varying amounts of phenolic compounds were also found in other similar studies [8,27], and this was also in agreement with the results of Karabagias et al. reported similar varying content (mg/kg) in quercetin, syringic acid, kaempferol, chrysin and myricetin for fir, pine, orange and thyme honey samples tested from the Greek market [6].

### 2.3. Multivariate Statistical Analysis

#### 2.3.1. Principal Component Analysis (PCA)

**Figure 2:**
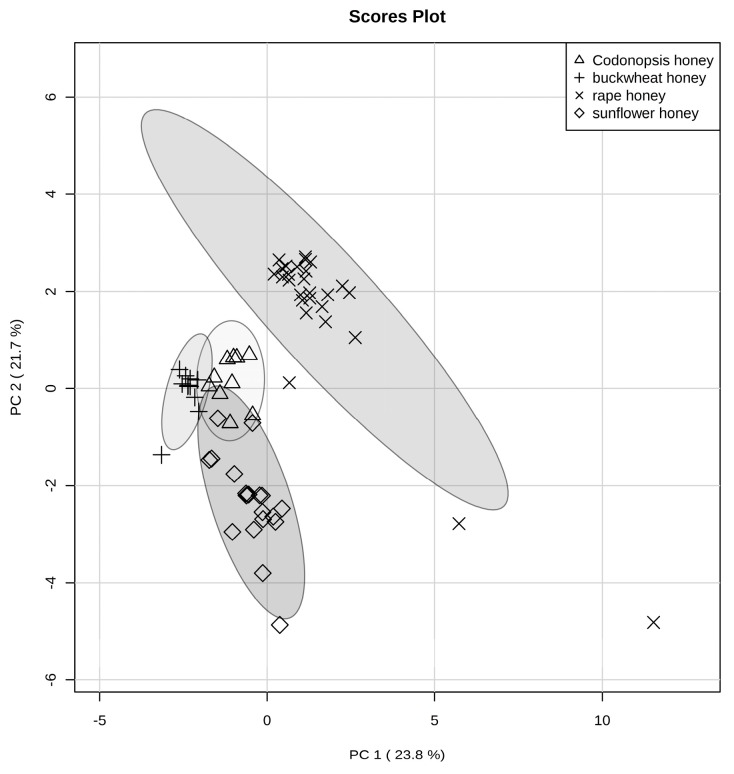
PCA score plot of rapeseed, buckwheat, sunflower, and *Codonopsis* honey samples.

Multivariate statistical analysis is an important feature of modern analytical approaches allowing characterization of complex matrices by extracting information from multivariate chemical data. Three honey samples (S17 and C10, C11) were exclude in the following statistical analysis according to the pollen analysis results. The proline and phenolic profiles of the four unifloral honeys were analyzed using PCA to investigate subtle differences for the different types of honey samples. The first three principal components (PCs) explained 60.6% of the total variance, where PC1 explained 23.8%, PC2 21.7% and PC3 15.1%, respectively. The scatter plot of the first two PCs (PC1 and PC2) for the classification of honey samples according to their botanical origin are shown in Figure 2. The 95% confidence interval is indicated by confidence ellipses for each set of honey samples in the PCA score plot (Figure 2). The rapeseed honey samples were well separated from the buckwheat, sunflower, and *Codonopsis* honey samples in the positive region of PC1. The other honey group was well clustered in the negative region of PC1. *Codonopsis* honeys were distributed around the origin of the score plot. The three clusters of buckwheat, sunflower and *Codonopsis* honey samples, overlapped partially. Sunflower honey samples were separated from rapeseed honey samples by PC2. The highest eigenvector in PC1 was explained by kaempferol, myricetin, quercetin, morin and protocatechuic, whilst the highest eigenvector in PC2 was explained by the benzoic acid, caffeic acid, galangin, chrysin and quercetin, whilst PC3 was explained by *p*-coumaric acid, *p*-hydroxybenzoic acid, rutin, caffeic acid and myricetin (Table 2).With respect to the different floral origins, the clustering of rapeseed, buckwheat, and sunflower honey, was separated from the other three honey samples, due to an obvious boundary in the PC1-PC2 grouping, respectively.

#### 2.3.2. Hierarchical Clustering (HCA)

**Figure 3:**
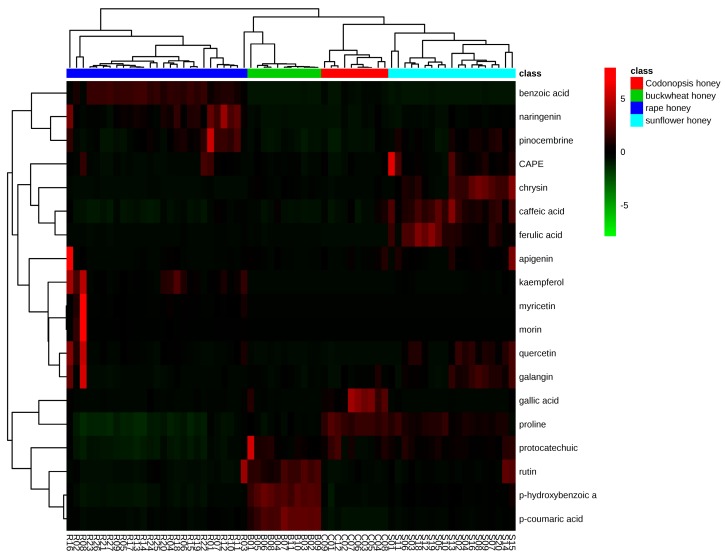
Hierarchical clustering and heatmap visualization of phenolic compounds in rapeseed, buckwheat, sunflower, and *Codonopsis* honey samples. Rows represent phenolic compound and columns represents honey samples. The data is the mean of three values for each sample. The data are normalized by rows using the function “scale”. Cells are colored based on concentrations in honey samples, red represent a high concentration and blue a low concentration. The row dendrogram indicate the correlation between groups of phenolic compounds; the column dendrogram indicates the correlation between different types of honey samples.

Hierarchical clustering was used to show the phenolic and proline differences of different floral origin honeys. The dendrograms of the hierarchical clustering and heatmap results are shown in Figure 3. Honeys samples were obviously clustered into two main groups. One group contained mainly rapeseed honeys and the other group included buckwheat, sunflower, and *Codonopsis* honeys, indicting similarities between these three types of honeys, which was consistent with the PCA results. All samples of the four honey types were arranged in homogenous clusters, indicating that different floral origin honeys may be distinguished based on their phenolic profiles and proline content.

The heatmap visualization of phenolic compounds revealed that buckwheat honey samples can be grouped together, showing high concentrations of *p*-hydroxybenzoic acid, *p*-coumaric acid, and rutin. The rapeseed honey samples were grouped together, and show high concentrations of benzoic acid, kaempferol, naringenin, and pinocembrin. The sunflower and *Codonopsis* honey samples showed high concentrations of galangin, CAPE, and caffeic acid, and were grouped closely. Sunflower and *Codonopsis* honey samples were positioned separately with a prominent level of quercetin and chrysin in sunflower honeys and relative high level of gallic acid in *Codonopsis* honeys.

### 2.4. Discrimination of Honey Samples Based on Linear Discriminate Analysis (LDA)

**Figure 4:**
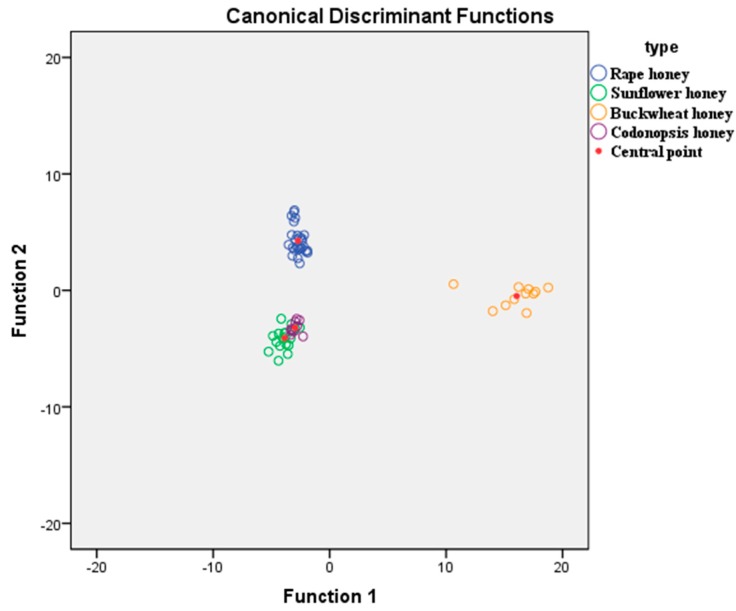
Botanical differentiation of four unifloral honeys based LDA on phenolic compounds and proline.

LDA was used for the categorization of the honey samples. The grouping variables were the sixty-seven honey samples of different types (rapeseed, sunflower, buckwheat and *Codonopsis*) and the independent variables were the 18 phenolic compounds and proline. Results showed that three statistically significant discriminant functions were formed (Wilk’s Lamda = 0, X2 = 462.611, df = 57, *p* value = 0 < 0.05) for the first function, (Wilk’s Lamda = 0.011, X2 = 244.139, df = 36, *p* value = 0 < 0.05) for the second and (Wilk’s Lamda = 0.176, X2 = 94.630, df = 17, *p* value = 0 < 0.05) for the third. The first discriminant function accounted for 73.8% of total variance while the second accounted for19.8%. Both accounted for 93.6% of the total variance. Figure 4 shows the scatter plot of honey samples defined by the discriminant functions. It was shown that the rapeseed and buckwheat honeys were well differentiated from sunflower and *Codonopsis* honeys. More specifically, the first discriminant function clearly separated buckwheat honey from all other honey types while the second discriminant function clearly separated rapeseed honey from all other honey types. The overall correct classification rate was 98.5% and 94.0% separately, using both the original and the cross validation method. Correct classification (100.0%) was obtained for *Codonopsis* honey samples followed by those of sunflower, rapeseed, and buckwheat (correct classification 94.7%, 92.6% and 90.9%, separately).

These results are in agreement with those of Zhao et al. who indicated that phenolic compounds could be used for the determination of floral origin [11]. And when conjunction with other physicochemical parameters, the predicted results will be more reliable by using multivariate statistical analysis [6,8,24].

## 3. Materials and Methods

### 3.1. Honey Samples and Pollen Analysis

**Figure 5:**
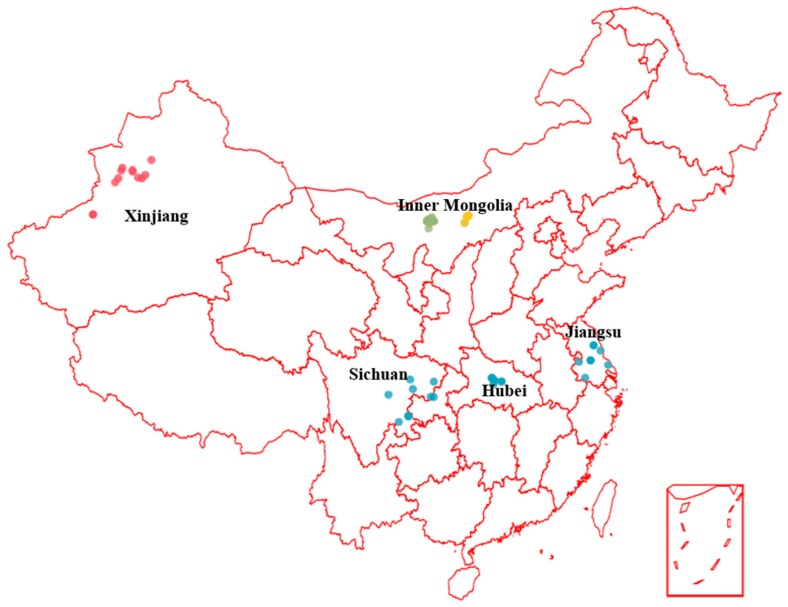
Collection locations of honey samples in the map of China. Blue dots, rapeseed honey; yellow dots, sunflower honey; green dots, buckwheat honey red dots, *Codonopsis* honey.

A total of seventy honey samples of four different floral types (rapeseed honeys, sunflower honeys, buckwheat honeys and *Codonopsis* honeys) were collected from Hubei, Jiangsu, Sichuan, Inner Mongolia and Xinjiang China (marked in Figure 5) in the flowering season of 2015. Honey samples were collected through beekeepers accompanied by our researcher. Honey samples were stored in the dark at 4–5 °C until analyzed. Information about season, hive location and available floral sources were collected by asking the beekeepers to ensure the authenticity of botanical origin. The detailed information about the honey samples is summarized in Supplementary Table S2. Moreover, their floral origins were confirmed by melissopalynological analysis [35]. Briefly, ten grams of each honey was dissolved in 20 mL of warm water (40 °C). The solution was centrifuged for 5 min at 4000 r/min, the supernatant solution was decanted, and the centrifugal step was repeated twice to remove excess water. The sediments were blended with glycerin. Two slides were prepared from each sample and photographed under a DM2500 light microscope (Leica, Heidelberg, Germany). Pollen types were identified by comparison with reference slides of pollen collected directly from the plants in the study and reference images of Pollen and Apicultural Plants in literature [36]. About 500 pollen grains were counted from each sample. The percentage frequency of the four characteristic pollen types in honey samples was calculated. Twenty-seven rapeseed honeys and eleven buckwheat honeys were classified as unifloral according to the melissopalynological analysis, with *Brassica campestris* L. pollen the share being in the range from 74 to 96%, and the *Fagopyrum esculentum* pollen share in the range from 66 to 96%, respectively; nineteen sunflower honey samples were unifloral with *Helianthus annuus* L. pollen in the range from 48 to 80%; and ten *Codonopsis* honey samples were unifloral *with Codonopsis pilosula* pollen shares in the range from 50 to 65% (Supplementary Table S2). Three honey samples (S17 and C10, C11) which were defined as sunflower honey and *Codonopsis* honey by the beekeepers, however, were classified as multifloral (characteristic pollen type <45%) [35,36], according to the pollen analysis, and were excluded from the statistical analysis.

### 3.2. Chemicals

Gallic (99%), protocatechuic (99%), *p*-hydroxybenzoic (99%), caffeic (98%), *p*-coumaric (98.0%) and ferulic acid (99.0%) were purchased from J & K Scientific (Beijing, China). Benzoic acid (99.5%) and flavonoids, i.e., rutin (95.0%), myricetin (96.0%), apigenin (95.0%), kaempferol (97.0%), quercetin (98.0%), morin (95.0%), pinocembrin (95.0%), naringenin (98.0%), chrysin (97.0%), galangin (95.0%) caffeic acid phenethyl ester (CAPE, 97.0%) and 4-fluoro-7-nitro-2,1,3-benzoxadiazole (NBD-F, purity ≥ 98.0%) were supplied by Sigma–Aldrich (St. Louis, MO, USA).

l-Proline (Pro) was supplied by Aladdin (Shanghai, China). Methanol and acetonitrile (HPLC grade) were purchased from MREDA (MREDA Technology Inc., Columbia, TN, USA). Ultrapure water was produced by using a Milli-Q system (Millipore, MA, USA). Stock solutions of single phenolic compounds (1~10 mg/mL) were prepared by dissolving appropriate amounts of phenolic standards in methanol. Mixed intermediate solutions were prepared by mixing of appropriate stock solutions and dissolving with methanol. Series of working standard solutions were diluted directly to the required concentrations with pure water on the day of use, based on the sensitivity of detection and the linearity range of the study.

### 3.3. Solid-Phase Extraction and HPLC–ESI-MS/MS Analysis

Solid-phase extraction (SPE) method was used to extract phenolic compounds from honey according to a previous method with slight modifications [15]. Honey samples (10.0 g) were mixed with 30 mL acidulated water (pH = 2.0) and vortexed to completely blend. The solution was then centrifuged for 10 min at 10,000 rpm to remove impurities. Prior to extraction, the Oasis HLB cartridge column (Waters, Wexford, Ireland) was activated with methanol (10 mL) and acidulated water (pH 2.0, 10 mL). After the supernatant was loaded, the column was then washed with distilled water and the phenolic compounds were eluted with methanol into a 10 mL flask. The solvent was evaporated to dryness at 40 °C and the residue was dissolved in 1.0 mL of 0.1% formic acid:acetonitrile (70:30, *v*/*v*). The sample was then mixed on a vortexer for 2 min and filtered through a filter membrane with 0.25μm pore size. All measurements were performed in triplicate.

**Figure 6:**
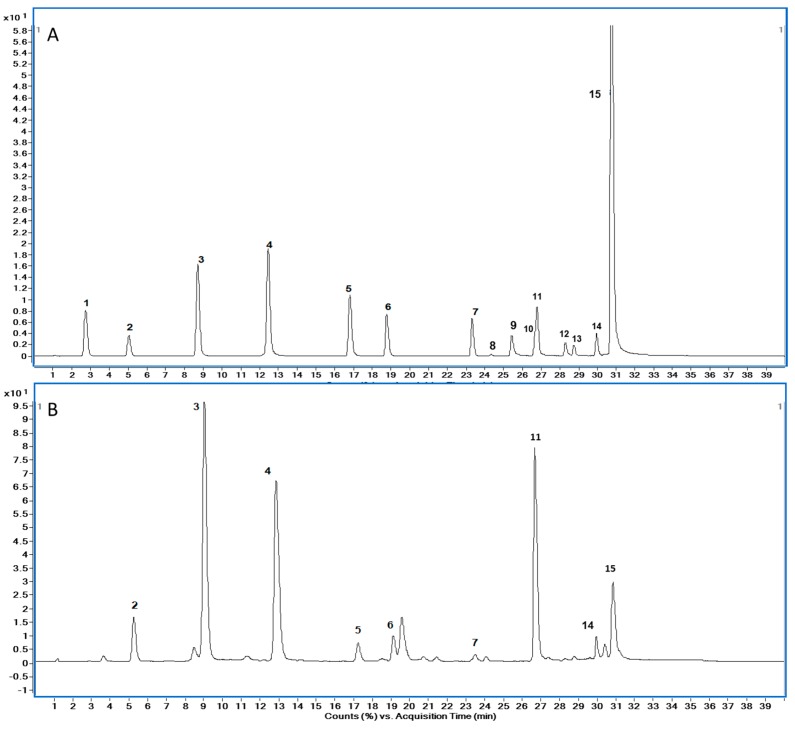
(**A**) TIC chromatogram of phenolic standard. (**B**) TIC chromatogram of buckwheat honey. Compound names and retention times (min) are as follows: (A): peak 1, gallic acid (2.74), peak 2, protocatechuic acid (5.05), peak 3, *p*-hydroxybenzoic acid (8.73), peak 4, caffeic acid (12.52), peak 5, *p*-coumaric acid (16.88), peak 6, ferulic acid (18.88), peak 6, benzoic acid (18.92), peak 7,rutin (23.42), peak 8, myricetin (24.41), peak 9, morin (25.47), peak 10, quercetin (26.70), peak 11, naringenin (26.82), peak 12, kaempferol (28.29), peak 13, apigenin (28.75), peak 14, pinocembrin (29.95), peak 15, CAPE (30.77), chrysin (30.95), galangin (31.11). (B): peak 2, protocatechuic acid (5.05), peak 3, *p*-hydroxybenzoic acid (8.73), peak 4, caffeic acid (12.52), peak 5, *p*-coumaric acid (16.88), peak 6, ferulic acid (18.88), peak 7, rutin (23.42), peak 11, naringenin (26.82), peak 14, pinocembrin (29.95), peak 15, CAPE (30.77), chrysin (30.95), galangin (31.11).

The extracts were analysed using an Agilent C18 column (100 mm × 2.1 mm, 2.7 μm; Agilent, Wilmington, DE, USA) within 50 min. The column temperature was maintained at 30°C. 0.1% formic acid in water (solvent A) and methanol (solvent B) were used as mobile phase, and the flow rate was 0.2 mL/min. The HPLC-MS conditions were as described in our previous study [15]. A typical TIC chromatogram from standard phenolic compounds and buckwheat honey sample is given in Figure 6A,B. The MS parameters used to measure phenolic acids and flavonoids and the transitions from precursor to product ions are shown in Supplementary Table S3. The identification and quantification of phenolic compounds was performed with MassHunter software (Agilent Technologies). The detailed description of the calibration curves is presented in Supplementary Table S4.

### 3.4. HPLC analysis of Proline

The extraction, derivatization and chromatographic separation method for proline in the honey samples were performed as described by Li et al. [37]. Briefly, twenty milliliters of 0.1 M boric acid buffer was added to 1.0 g of honey and subject to ultrasound treatment for approximately 10 min. After extraction, the final volume was diluted to 50 mL with borate buffer solution (pH = 8.0). The solution of honey was filtered through 0.25 μm nylon filter membrane and transferred to vials for refrigerator storage prior to derivatisation. All measurements were performed in triplicate.

### 3.5. Data Statistical Analysis

Principal component analysis (PCA), Hierarchical clustering (HCA) were performed using MetaboAnalyst 2.0 through the ‘Statistical Analysis’ tool [38]. Prior to statistical analysis, all data were normalized using ‘Autoscaling’ in the MetaboAnalyst program in order to prevent a dominating effect of more highly abundant components over components present in much smaller quantities. Unsupervised Hierarchical clustering using Ward’s method of agglomeration and Pearson distances to evaluate the similarity between samples was used to provide an overview on clustering of different floral origin honeys. Comparison of the means was achieved by a one-way analysis of variance (ANOVA). Linear Discriminant Analysis (LDA) was applied to explore the possibility of classification of honey samples according to floral origin. ANOVA and LDA analysis was performed using the SPSS 20.0 statistical software (International Business Machines Corporation, Armonk, NY, USA).

## 4. Conclusions

This research study characterized the phenolic and proline profiles of four types of unifloral honey (rapeseed, sunflower, buckwheat, and *Codonopsis* honey) collected from beekeepers in China. The proline and phenolic content showed significant differences in the four types of honey. Buckwheat honey contained the highest content of proline, *p*-coumaric acid, and *p*-hydroxybenzonic acid; rapeseed honey has the lowest content of proline, and highest content of benzoic acid. PCA and hierarchical clustering results showed that rapeseed honey could be separated from buckwheat, sunflower and *Codonopsis* honey by the selected phenolic compounds and proline. LDA results showed that phenolic compounds and proline in combination with chemometrics may differentiate the floral origin of four Chinese honeys with a classification rate of 94.0%. On the other hand, the determination of chemical composition of Chinese unifloral honey, could lead to the formation of a database in which can be used to classify honeys from different sources.

## Figures and Tables

**Table 1:** Content Range of Phenolic Compounds in the Four Types of Unifloral Honey (mg/kg).

Compounds	Rapeseed Honey (*n* = 27)			Sunflower Honey (*n* = 19)			Buckwheat Honey (*n* = 11)			Codonopsis Honey (*n* = 10)		
	Max	Min	Mean ± SD	Max	Min	Mean ± SD	Max	Min	Mean ± SD	Max	Min	Mean ± SD
Gallic acid	0.080	0.003	0.015 ± 0.018 b	0.088	0.002	0.011 ± 0.020 b	0.324	0.001	0.039 ± 0.086 b	0.3	0.012	0.157 ± 0.103 a
Protocatechuic acid	0.118	0.033	0.066 ± 0.021 b	0.262	0.082	0.133 ± 0.050 b	1.314	0.017	0.361 ± 0.324 a	0.198	0.042	0.096 ± 0.051 b
*p*-Hydroxybenzoic acid	1.220	0.431	0.726 ± 0.169 b	1.912	0.105	0.939 ± 0.369 b	11.174	0.067	7.967 ± 3.764 a	0.715	0.376	0.513 ± 0.111 b
Caffeic acid	0.465	0.015	0.122 ± 0.095 b	1.023	0.017	0.460 ± 0.195 a	0.872	0.004	0.202 ± 0.223 b	0.348	0.015	0.145 ± 0.110 b
*p*-Coumaric acid	0.323	0.101	0.188 ± 0.054 b	0.272	ND	0.138 ± 0.067 b	3.188	0.022	2.101 ± 1.061 a	0.051	0.002	0.017 ± 0.019 b
Ferulic acid	0.066	0.013	0.032 ± 0.015 b	0.449	0.067	0.217 ± 0.114 a	0.100	0.016	0.038 ± 0.026 b	0.01	ND	0.005 ± 0.003 b
Benzoic acid	18.108	1.800	9.475 ± 4.479 a	0.176	0.006	0.097 ± 0.031 b	0.478	0.018	0.228 ± 0.121 b	1.369	0.078	0.611 ± 0.364 b
Rutin	0.248	ND	0.077 ± 0.049 b	0.187	ND	0.049 ± 0.048 bc	0.304	0.159	0.225 ± 0.095 a	0.018	ND	0.018 ± 0.005 c
Myricetin	0.507	ND	0.116 ± 0.097	ND	ND	ND	ND	ND	ND	ND	ND	ND
Morin	11.115	ND	0.607 ± 2.133 a	0.007	ND	0.004 ± 0.002 a	0.015	ND	0.015 ± 0.004 a	ND	ND	ND
Quercetin	5.184	0.022	0.402 ± 0.989 a	0.659	0.002	0.280 ± 0.215 a	0.154	0.001	0.046 ± 0.043 a	0.016	0.004	0.092 ± 0.039 a
Naringenin	0.032	0.001	0.013 ± 0.008 a	ND	ND	ND	ND	ND	ND	0.004	0.002	0.003 ± 0.001 b
Kaempferol	1.897	0.012	0.214 ± 0.367 a	0.077	ND	0.026 ± 0.019 a	0.026	0.011	0.019 ± 0.008 a	0.019	0.008	0.014 ± 0.006 a
Apigenin	0.036	ND	0.009 ± 0.007 a	0.015	ND	0.004 ± 0.003 a	0.006	ND	0.003 ± 0.002 a	0.005	ND	0.003 ± 0.002 a
Pinocembrin	0.119	ND	0.046 ± 0.030 a	0.029	ND	0.024 ± 0.013 b	0.035	ND	0.035 ± 0.010 c	0.024	ND	0.024 ± 0.007 c
CAPE	0.087	ND	0.013 ± 0.021 a	0.059	ND	0.016 ± 0.014 a	0.026	ND	0.014 ± 0.008 a	0.027	ND	0.008 ± 0.008 a
Chrysin	0.096	ND	0.035 ± 0.021 b	1.384	ND	0.700 ± 0.461 a	0.762	0.016	0.277 ± 0.208 b	0.175	ND	0.059 ± 0.057 b
Galangin	0.329	ND	0.194 ± 0.064 a	0.042	ND	0.034 ± 0.014 b	ND	ND	ND	ND	ND	ND

Results in the same row with the different lowercase were significantly different (Duncan test, *p* < 0.05). Three measurements were performed for each sample. CAPE, Caffeic acid phenethyl ester; SD, Standard Deviation. ND, not detected.

**Table 2:** Loadings of the variables for the first three PCs.

Variables	PC1	PC2	PC3
Kaempferol	0.856	0.089	0.258
Myricetin	0.722	0.247	0.435
Quercetin	0.713	0.589	0.182
Morin	0.702	0.266	0.433
Protocatechuic acid	−0.509	0.509	0.109
Benzoic acid	0.457	−0.802	−0.143
Caffeic acid	−0.147	0.729	−0.469
Galangin	0.607	0.715	0.146
Chrysin	−0.084	0.7	−0.194
Ferulic acid	−0.145	0.56	−0.396
Proline	−0.435	0.519	−0.392
CAPE	0.136	0.462	−0.225
*p*-Coumaric acid	−0.454	0.051	0.752
*p*-Hydroxybenzoic acid	−0.546	0.214	0.692
Rutin	−0.372	0.216	0.652
Gallic acid	−0.19	0	−0.164
Pinocembrin	0.355	0.004	−0.403
Naringenin	0.535	−0.32	−0.244
Apigenin	0.294	0.447	−0.052

The compounds with high contribution values for each PCs were highlighted in red color.

## Supplementary Materials

The following are available online: Table S1: Content of phenolic compounds and proline in the three multifloral honey samples (mg/kg), Table S2: Description of Sampling Regions and characteristic pollen type frequency for different types of honey in China, Table S3: The mass spectrum parameters applied for phenolic compounds, Table S4: Regression equation, r2, Linear range, limit of detection (LOD) and limit of quantification (LOQ) of phenolic compounds.
