# Supplementary material for: Characterization of Chinese Unifloral Honeys Based on Proline and Phenolic Content as Markers of Botanical Origin, Using Multivariate Analysis
Source: Molecules. 2017 May 17;22(5):735. doi: 10.3390/molecules22050735 (PMC6154540; doi:10.3390/molecules22050735)
Supplement: Supplementary file 1 [file molecules-22-00735-s001.pdf]

Table S1 Content of phenolic compounds and proline in the three mutifloral honey samples (mg/ kg).

Compounds	S17	C10	C11
Gallic acid	0.007±0.000	0.012±0.000	0.063±0.003
Protocatechuic acid	0.255±0.001	0.081±0.001	0.209±0.007
p-Hydroxybenzoic acid	0.119±0.000	0.771±0.009	0.400±0.076
Caffeic acid	0.277±0.006	0.068±0.001	0.029±0.012
p-Coumaric acid	0.203±0.008	0.310±0.001	0.063±0.000
Ferulic acid	0.086±0.001	0.174±0.005	0.007±0.002
Benzoic acid	0.100±0.011	0.231±0.012	0.169±0.000
Rutin	0.058±0.028	ND	ND
Myricetin	ND	ND	ND
Morin	ND	ND	ND
Quercetin	0.185±0.000	0.129±0.003	0.396±0.007
Naringenin	ND	ND	ND
Kaempferol	0.0020±0.000	0.091±0.001	0.087±0.020
Apigenin	0.001±0.000	0.002±0.000	0.001±0.000
Pinocembrine	ND	ND	ND
CAPE	0.007±0.000	0.004±0.000	ND
Chrysin	0.278±0.008	ND	ND
Galangin	ND	ND	ND
Proline	612.55±58.60	497.74±1.01	219.77±3.66

Three measurements were performed for each sample. CAPE, Caffeic acid phenethyl ester; ND, not detected

Table S2

Description of sampling regions, sampleing time and characteristic pollen type frequency for different types of honey in China.

Sample ID	Honey type	Sampling month	Dominate pollen type(frequence)	Place of production	location coordinates(N,E)
R01	Rape honey	2015.03.25	*Brassica campestris* L. (75%)	Dazhou city, Sichuan province	31.18,107.42
R02	Rape honey	2015.03.26	*Brassica campestris* L. (92%)	Guangan city, Sichuan province	30.27,106.95
R03	Rape honey	2015.03.27	*Brassica campestris* L. (85%)	Suining city, Sichuan province	30.74,105.76
R04	Rape honey	2015.03.26	*Brassica campestris L.* (93%)	Guangan city, Sichuan province	30.28,107.17
R05	Rape honey	2015.03.26	*Brassica campestris* L. (85%)	Yibin city, Sichuan province	28.77,104.63
R06	Rape honey	2015.03.24	*Brassica campestris* L. (87%)	Luzhou city, Sichuan province	29.09,105.41
R07	Rape honey	2015.03.27	*Brassica campestris* L. (79%)	Mianyang city, Sichuan province	31.31,105.54
R08	Rape honey	2015.03.24	*Brassica campestris* L. (84%)	Luzhou city, Sichuan province	29.15,105.38
R09	Rape honey	2015.03.25	*Brassica campestris* L. (90%)	Chengdu city, Sichuan province	30.40,103.81
R10	Rape honey	2015.03.24	*Brassica campestris* L. (81%)	Zhongxiang city, Hubei province	31.19,112.83
R11	Rape honey	2015.03.23	*Brassica campestris* L. (93%)	Zhongxiang city, Hubei province	31.19,112.83
R12	Rape honey	2015.03.25	*Brassica campestris* L. (87%)	Jingmen city, Hubei province	31.40,112.05
R13	Rape honey	2015.03.27	*Brassica campestris* L. (77%)	Jingmen city, Hubei province	31.40,112.05
R14	Rape honey	2015.03.26	*Brassica campestris* L. (83%)	Jingmen city, Hubei province	31.40,112.05
R15	Rape honey	2015.03.23	*Brassica campestris* L. (92%)	Jingmen city, Hubei province	31.26,112.22
R16	Rape honey	2015.03.22	*Brassica campestris* L. (86%)	Jingmen city, Hubei province	31.26,112.22
R17	Rape honey	2015.03.26	*Brassica campestris* L. (74%)	Jingmen city, Hubei province	31.26,112.22
R18	Rape honey	2015.03.22	*Brassica campestris* L. (80%)	Jingmen city, Hubei province	31.26,112.22
R19	Rape honey	2015.03	*Brassica campestris* L. (81%)	Jingmen city, Hubei province	31.04,112.19
R20	Rape honey	2015.04.23	*Brassica campestris* L. (94%)	Changzhou city, Jiangsu province	31.41,119.49
R21	Rape honey	2015.04.23	*Brassica campestris* L. (84%)	Nantong city, Jiangsu province	32.18,121.32

R22	Rape honey	2015.04.20	*Brassica campestris* L. (74%)	Dafeng city, Jiangsu province	33.03,120.71
R23	Rape honey	2015.04.22	*Brassica campestris* L. (77%)	Yancheng city, Jiangsu province	33.35,120.16
R24	Rape honey	2015.04.22	*Brassica campestris* L. (96%)	Taizhou city, Jiangsu province	32.46,119.92
R25	Rape honey	2015.04.25	*Brassica campestris* L. (92%)	Taizhou city, Jiangsu province	32.46,119.92
R26	Rape honey	2015.04.20	*Brassica campestris* L. (95%)	Dongtai city, Jiangsu province	32.37,118.99
R27	Rape honey	2015.08.12	*Brassica campestris* L. (92%)	Yancheng city, Jiangsu province	33.35,120.16
S01	Sunflower honey	2015.08.12	*Helianthus annuus* L. (48%)	Hangjinhouqi, Bayan Nur City, Inner Mongolia Autonomous Region	40.65,107.09
S02	Sunflower honey	2015.08.12	*Helianthus annuus* L. (79%)	Hangjinhouqi, Bayan Nur City, Inner Mongolia Autonomous Region	40.78,106.88
S03	Sunflower honey	2015.08.12	*Helianthus annuus* L. (50%)	Hangjinhouqi, Bayan Nur City, Inner Mongolia Autonomous Region	40.90,107.15
S04	Sunflower honey	2015.08.12	*Helianthus annuus* L. (65%)	Hangjinhouqi,Bayan Nur City, Inner Mongolia Autonomous Region	40.87,106.97
S05	Sunflower honey	2015.08.12	*Helianthus annuus* L. (57%)	Bayan Nur City, Inner Mongolia Autonomous Region	40.34,107.02
S06	Sunflower honey	2015.08.12	*Helianthus annuus* L. (74%)	Hangjinhouqi,Bayan Nur City, Inner Mongolia Autonomous Region	40.78,106.88
S07	Sunflower honey	2015.08.12	*Helianthus annuus* L. (61%)	Hangjinhouqi, Bayan Nur City, Inner Mongolia Autonomous Region	40.78,106.88
S08	Sunflower honey	2015.08.12	*Helianthus annuus* L. (64%)	Linhe district, Bayan Nur City, Inner Mongolia Autonomous Region	40.67,109.87
S09	Sunflower honey	2015.08.12	*Helianthus annuus* L. (59%)	Hangjinhouqi, Bayan Nur City, Inner Mongolia Autonomous Region	40.90,107.15
S10	Sunflower honey	2015.08.12	*Helianthus annuus* L. (80%)	Hangjinhouqi, Bayan Nur City, Inner Mongolia Autonomous Region	40.78,106.88
S11	Sunflower honey	2015.08.12	*Helianthus annuus* L. (50%)	Hangjinhouqi, Bayan Nur City, Inner Mongolia Autonomous Region	40.87,106.97
S12	Sunflower honey	2015.08.12	*Helianthus annuus* L. (66%)	Linhe district, Bayan Nur City, Inner Mongolia Autonomous Region	41.00,107.27
S13	Sunflower honey	2015.08.12	*Helianthus annuus* L. (77%)	Hangjinhouqi, Bayan Nur City, Inner Mongolia Autonomous Region	40.76,107.01
S14	Sunflower honey	2015.08.12	*Helianthus annuus* L. (73%)	Linhe district, Bayan Nur City, Inner Mongolia Autonomous Region	40.90,107.15
S15	Sunflower honey	2015.08.12	*Helianthus annuus* L. (57%)	Linhe district, Bayan Nur City, Inner Mongolia Autonomous Region	40.90,107.15
S16	Sunflower honey	2015.08.12	*Helianthus annuus* L. (53%)	Hangjinhouqi, Bayan Nur City, Inner Mongolia Autonomous Region	40.77,107.42
S17	Mutifloral honey	2015.08.12	*Helianthus annuus* L. (28%)	Linhe district, Bayan Nur City, Inner Mongolia Autonomous Region	40.90,107.15
S18	Sunflower honey	2015.08.12	*Helianthus annuus* L. (69%)	Hangjinhouqi, Bayan Nur City, Inner Mongolia Autonomous Region	40.77,107.42
S19	Sunflower honey	2015.08.12	*Helianthus annuus* L. (77%)	Hangjinhouqi, Bayan Nur City, Inner Mongolia Autonomous Region	40.77,107.42
S20	Sunflower honey	2015.08.11	*Helianthus annuus* L. (73%)	Hangjinhouqi, Bayan Nur City, Inner Mongolia Autonomous Region	40.77,107.42
B01	Buckwheat honey	2015.08.11	*Fagopyrum esculentum* (86%)	Guyang County, Baotou city, Inner Mongolia Autonomous Region	41.10,110.17

B02	Buckwheat honey	2015.08.11	*Fagopyrum esculentum* (92%)	Guyang County, Baotou city, Inner Mongolia Autonomous Region	41.10,110.17
B03	Buckwheat honey	2015.08.11	*Fagopyrum esculentum* (75%)	Guyang County, Baotou city, Inner Mongolia Autonomous Region	41.10,110.17
B04	Buckwheat honey	2015.08.11	*Fagopyrum esculentum* (86%)	Guyang County, Baotou city, Inner Mongolia Autonomous Region	41.10,110.17
B05	Buckwheat honey	2015.08.11	*Fagopyrum esculentum* (86%)	Guyang County, Baotou city, Inner Mongolia Autonomous Region	41.10,110.17
B06	Buckwheat honey	2015.08.14	*Fagopyrum esculentum* (66%)	Wuchuan County, Baotou city, Inner Mongolia Autonomous Region	40.67,109.89
B07	Buckwheat honey	2015.08.14	*Fagopyrum esculentum* (80%)	Guyang County, Baotou city, Inner Mongolia Autonomous Region	41.10,110.17
B08	Buckwheat honey	2015.08.14	*Fagopyrum esculentum* (84%)	Guyang County, Baotou city, Inner Mongolia Autonomous Region	41.10,110.17
B09	Buckwheat honey	2015.08.11	*Fagopyrum esculentum* (90%)	Guyang County, Baotou city, Inner Mongolia Autonomous Region	41.10,110.17
B10	Buckwheat honey	2015.08.11	*Fagopyrum esculentum* (93%)	Guyang County, Baotou city, Inner Mongolia Autonomous Region	41.10,110.17
B11	Buckwheat honey	2015.08	*Fagopyrum esculentum* (96%)	Guyang County, Baotou city, Inner Mongolia Autonomous Region	41.04,110.07
C01	Codonopsis honey	2015.08	*Codonopsis pilosula* (50%)	Nileke County, Xinjiang Uyghur Autonomous Region	43.82,83.41
C02	Codonopsis honey	2015.08	*Codonopsis pilosula* (52%)	Nileke County, Xinjiang Uyghur Autonomous Region	43.97,82.61
C03	Codonopsis honey	2015.08	*Codonopsis pilosula* (52%)	Nileke County, Xinjiang Uyghur Autonomous Region	43.80,82.52
C04	Codonopsis honey	2015.08	*Codonopsis pilosula* (60%)	Nileke County, Xinjiang Uyghur Autonomous Region	43.73,83.44
C05	Codonopsis honey	2015.08	*Codonopsis pilosula* (53%)	Nileke County, Xinjiang Uyghur Autonomous Region	43.54,84.44
C06	Codonopsis honey	2015.08	*Codonopsis pilosula* (62%	Xinyuan County, Xinjiang Uyghur Autonomous Region	43.38,83.87
C07	Codonopsis honey	2015.08	*Codonopsis pilosula* (59%)	Nileke County, Xinjiang Uyghur Autonomous Region	43.10,82.03
C08	Codonopsis honey	2015.08	*Codonopsis pilosula* (64%)	Gouliu County, Nileke County, Xinjiang Uyghur Autonomous Region	43.34,82.29
C09	Codonopsis honey	2015.08	*Codonopsis pilosula* (51%)	Xinyuan County, Xinjiang Uyghur Autonomous Region	43.34,84.18
C10	Mutifloral honey	2015.08	*Codonopsis pilosula* (20%)	Yili Autonomous Prefecture, Xinjiang Uyghur Autonomous Region	44.43,84.92
C11	Mutifloral honey	2015.08	*Codonopsis pilosula* (18%)	Xinjiang Uyghur Autonomous Region	41.17,80.27
C12	Codonopsis honey	2015.08	*Codonopsis pilosula* (55%)	Xinjiang Uyghur Autonomous Region	41.17,80.27

Table S3

The mass spectrum parameters applied for phenolic compounds.

Compounds	CAS	Precursor ion (m/z)	Product Ion (m/z)	Dwell time (ms)	Fragmentor(v)	CE(eV)
Gallic acid	149-91-7	168.7	125	12	98	8
Protocatechuic acid	99-50-3	153	109	12	95	10
p-Hydroxybenzoic acid	99-96-7	137	93.1	12	90	10
Caffeic acid	331-39-5	179	135	12	95	10
			107	12	95	22
p-Coumaric acid	501-98-4	162.7	118.7	12	85	8
Ferulic acid	1135-24-6	193	134	12	76	8
			178	12	76	4
Benzoic acid	65-85-0	121	77.1	12	75	5
Rutin	153-18-4	609	301	12	180	30
			271	12	180	45
Myricetin	529-44-2	317	151	12	150	25
			179	12	150	16
Morin	480-16-0	301	151	12	128	16
			125	12	128	16
Quercetin	117-39-5	301	151	12	150	16
			179	12	150	12
Naringenin	480-41-1	271	151	12	102	12
			119	12	102	24
Kaempferol	520-18-3	285	93	12	150	30
			117	12	150	40
Apigenin	520-36-5	269	117	12	128	30
			151	12	128	20
Pinocembrine	480-39-7	255	151	12	125	16

			213	12	125	12
CAPE	104594-70-9	283	135	12	150	20
			179	12	150	12
Chrysin	480-40-0	253	63	12	128	30
			143	12	128	30
Galangin	548-83-4	269	169	12	128	30
			171	12	128	20

CAPE, Caffeic acid phenethyl ester

Table S4

Regression equation, r2, linear range, limit of detection (LOD) and limit of quantification (LOQ) of phenolic compounds.

compounds	Regression qequation	r^2	Linear range (ng/mL)	LOD (ng/mL)	LOQ(ng/mL)
Gallic acid	y=133.283031*x	0.9975	16~2000	4.68	15.61
Protocatechuic acid	y=386.010656*x	0.9979	3.2~10000	5.24	17.47
p-Hydroxybenzoic acid	y=315.651183*x	0.9983	16~2000	5.66	18.87
Caffeic acid	y=491.858145*x	0.9762	16~10000	4.35	14.5
p-Coumaric acid	y=146.362096*x	0.9862	16~10000	2.73	9.11
Ferulic acid	y=107.635157*x	0.9998	16~10000	5.14	17.12
Benzoic acid	y=9.393531*x	0.9821	16~10000	118.81	396.04
Rutin	Y=53.568698*x	0.9996	6.4~4000	1.5	4.98
Myricetin	Y= 67.318600*x	1	200~5000	10.34	34.46
Morin	Y= 46.0177*x	0.9988	160~20000	5.93	18.39
Quercetin	Y=365.6770*x	0.9982	1.6~5000	1.53	5.12
Naringenin	Y=1225.2345*x	0.9889	1.6~200	0.09	0.31
Kaempferol	Y= 96.8538*x	0.9897	6.4~800	1.18	3.92
Apigenin	Y= 1352.600501*x	0.9891	0.32~200	0.11	0.38
Pinocembrine	Y=618.595701*x	0.995	1.6~200	0.43	0.95
CAPE	y = 3261.8x - 10084	0.9987	16~400	3.64	12.14
Chrysin	Y=16.997118*x	1	40~5000	0.08	0.28
Galangin	y = 98.558x + 512.03	0.9889	1.6~200	1.5	5.71

CAPE, Caffeic acid phenethyl ester
